# A Facile Synthesis of Highly Nitrogen-Doped Carbon Dots for Imaging and Detection in Biological Samples

**DOI:** 10.1155/2018/7890937

**Published:** 2018-07-15

**Authors:** Qianchun Zhang, Siqi Xie, Yanqun Yang, Yun Wu, Xingyi Wang, Jincheng Wu, Li Zhang, Junyu Chen, Yuan Wang

**Affiliations:** School of Biology and Chemistry, Xingyi Normal University for Nationalities, Xingyi 562400, China

## Abstract

A facile, green, and high-output hydrothermal synthesis was proposed for the fabrication of highly fluorescent nitrogen-doped carbon quantum dots (N-doped CDs). The nitrogen content in N-doped CDs reached 19.2% and demonstrated strong blue fluorescence emission was obtained with fluorescence quantum yield (QY) of up to 32.9%, which exhibit high fluorescence quantum yield, high photostability, and excellent biocompatibility. The N-doped CDs possess high photostability, low toxicity, and excellent biocompatibility, based on which the N-doped CDs were successfully applied as a fluorescence probe for cell imaging. Moreover, it was then successfully demonstrated for sensitive and selective detection of Fe^3+^ in serum.

## 1. Introduction

During the past few years, fluorescent CDs have attracted tremendous attention due to their unique optical and electronic properties [1–6]. Compared to conventional semiconductor quantum dots and organic fluorescent dyes, CDs possess several superior features including functionalization, low toxicity, excellent water dispersibility, tunable fluorescence emission, excellent photostability, upconversion, and biocompatibility, thus demonstrating potential application in the fields of bioimaging, in vivo theranostics, drug delivery, light-emitting diodes, photocatalysis solar cells, and heavy metal ion detection [7–11]. In addition, doping CDs with other nonmetallic components, such as N, S, and P, can inject electrons into carbon-based materials and change the electronic transport properties and PL properties [12, 13]. However, in most cases, the QY of the as-synthesized CD was less than 10%, and the QY is a key parameter to evaluate the quality of CDs, which limit the sensitivity and selectivity. So, synthesis of high-fluorescence carbon quantum dots is the direction of development.

The use of N-containing precursors has proved to be an effective route for obtaining N-doped CDs. Chen et al. [14] used 2-azidoimidazole as precursor in a hydrothermal process at 70°C overnight to obtain nitrogen-rich CDs. Lv et al. [15] using ethanediamine and citric acid as precursors obtained N-doped CDs and achieved good results in iron detection. Wang and Zhou. [16] used milk to prepare N-CDs hydrothermally at 180°C for 2 h. In another study, Hsu and Chang [17] found that compounds containing both amino and carboxyl groups are beneficial for synthesizing CDs with high PL quantum yield. Based on the benefits of N-doping in carbon nanostructures, it can be extrapolated that the introduction of N to carbon dots would further enhance their versatile properties. However, most N-doped CDs are unsatisfactory due to harsh synthetic conditions and long reaction times. Thus, a time-saving and eco-friendly synthesis of N-doped CDs is of interest.

Herein, a facile, green, and high-output thermal strategy is proposed for the fabrication of highly fluorescent N-doped CDs. We used L-citrulline as the precursor for a facile and eco-friendly one-step hydrothermal method without the assistance of any chemicals (except pure water) to obtain highly fluorescent N-doped CDs. The as-prepared N-doped CDs exhibit good water solubility, good biocompatibility, and high fluorescence quantum yield (32.9%). Owing to the unique properties of the N-doped CD nanoprobe with good membrane permeability and excellent biocompatibility, it was used for imaging of HeLa cells with high discrimination. Moreover, it was further applicated for detection of Fe^3+^ ions in serum, and the fluorescence intensity exhibited a good linear relationship in the Fe^3+^ concentration range from 0 to 50 *µ*M with a detection limit of about 37 nM.

## 2. Experimental

### 2.1. Materials

L-citrulline (98%) and quinine sulphate (98%) were purchased from J&K Scientific Inc. (Beijing, China). 3-(4,5-Dimethylthiazol-2-yl)-2, 5-diphenyltetrazolium bromide (MTT, 98%) was obtained from Sangon Biotechnology Inc. (Shanghai, China). Dimethyl sulphoxide (DMSO) was obtained from Xilong Reagents Company (Guangdong, China). Penicillin-streptomycin, Dulbecco's modified Eagle's medium (DMEM), and foetal bovine serum (FBS) were purchased from Solarbio (Beijing, China). NaCl, KCl, MgCl_2_, AlCl_3_, CaCl_2_, Cr(NO_3_)_3_, FeCl_2,_ FeCl_3_, Co(NO_3_)_2_, CuSO_4_, ZnCl_2_, Cd(NO_3_)_2_, SrCl_2_, and Hg(NO_3_)_2_ were purchased from Aladdin (Shanghai, China). HeLa cell lines were obtained from Cellcook. Human serum samples were provided by Xing Ying People's Hospital Blood Center (Xingyi, China). Ultrapure water (18.2 MΩ, Millipore Co., USA) was used in all experiments. Other chemical reagents (analytical grade) were purchased from Beijing Chemical Company (Beijing, China).

### 2.2. Instrumentation and Characterization

The morphologies and sizes of N-doped CDs were characterized by high-resolution transmission electron microscopy (HRTEM, Hitachi-F20) at an accelerating voltage of 200 kV and atomic force microscopy (AMF, Bruker Multimode 8) in the tapping mode. Fourier transform infrared (FT-IR) spectra were obtained on a Nicolet 6700 FT-IR spectrometer using KBr pellets. X-ray photoelectron spectroscopy (XPS) was performed using an ESCALAB 250Xi (Thermo Scientific). X-ray diffraction (XRD) was carried out using a Rigaku diffractometer in the 2*θ* range 10–80° with step width of 0.02°. UV-Vis absorption spectra were recorded on a DU 800 UV-Vis spectrophotometer. The PL decay curves were obtained on a Leica SP5 FLIM system using a 405 nm laser excitation source. Fluorescence spectroscopy and stability were measured on a PerkinElmer LS 55 with 5/5 nm slit width and equipped with a 1 cm quartz cell. A TGL-20LM-B high-speed refrigerated centrifuge (Hunan Xingke Instrument Co., Ltd., China) was used to purify the N-doped CDs. Cell imaging was carried out using a Leica SP8 confocal laser scanning microscope (Leica, Germany).

### 2.3. Synthesis of N-Doped CDs

N-doped CDs were synthesized by a facile hydrothermal method. Briefly, 0.50 g·L-citrulline was dissolved in 25 mL ultrapure water and subjected to ultrasonic oscillation for 20 min. The solution was transferred to a Teflon-equipped stainless steel autoclave and reacted at 220°C for 12 h. After the reaction liquid was cooled to room temperature, the reaction liquid was centrifuged at 17,000 rpm for 40 min to separate aggregated particles. The supernatant fluid was removed by filtration with a 0.22 *μ*m filter membrane. The as-prepared N-doped CD solution was stored at 4°C for further use.

### 2.4. MTT Assay and Intracellular Fluorescence Imaging

Cytotoxicity of the N-doped CDs was investigated with the cancer cell line HeLa by an MTT assay. HeLa cells were seeded in a 96-well plate at a density of 4 × 10^3^ cells per well for 24 h in an incubator (37°C, 5% CO_2_). The culture medium was replaced with 100 *μ*L fresh DMEM containing the N-doped CDs at concentrations of 0, 25, 50,100, 250, and 500 *μ*g/mL and incubated in an incubator (37°C, 5% CO_2_). After 24 or 48 h, 20 *µ*L MTT (5 mg/mL) solution was added to each cell well, which was incubated for 4 h. Subsequently, the culture medium with MTT was removed and 100 *µ*L DMSO was added, followed by shaking for 10 min. The optical density (OD) of each well at 490 nm was measured on the enzyme-linked immunosorbent detector.

HeLa cells (1 × 10^5^ cells/dish) were seeded in a confocal dish with 100 *μ*L fresh DMEM containing 10% FBS and incubated in an incubator (37°C, 5% CO_2_). After 24 h, the N-doped CDs with concentrations of 50, 200, 400, and 600 *µ*g/mL were added to the confocal dish and incubated at 37°C in 5% CO_2_ for another 4 h. Subsequently, the adherent cells were carefully washed three times with PBS (0.01 M, pH 7.4). Finally, the laser confocal microscopy imaging of HeLa cells was performed at excitations of 405 and 488 nm. Bright-field images were captured to ensure the locations of fluorescent tag signals.

### 2.5. Metal Ion Detection by N-Doped CDs

Sources of various metal ions such as Na^+^, Mg^2+^, Al^3+^, Ca^2+^, Cr^3+^, Fe^2+^, Fe^3+^, Co^2+^, Cu^2+^, Zn^2+^, Cd^2+^, Sr^2+^, K^+^, and Hg^2+^ were applied for detection. N-doped CD stock solution (2.0 mg/mL, 250 *μ*L) was mixed with 50 *μ*L of 120 *μ*M solutions of different metal ions to reach the final concentration of 20 *μ*M, respectively. Afterward, the mixtures were recorded under excitation at 360 nm. To evaluate the selectivity of this N-doped CD toward Fe^3+^, interference assays were performed under identical conditions using above ions and Fe^3+^, and the N-doped CD stock solution was added to different concentrations of Fe^3+^ solution in a similar manner.

## 3. Results and Discussion

### 3.1. Characterization of N-Doped CDs

**Figure 1:**
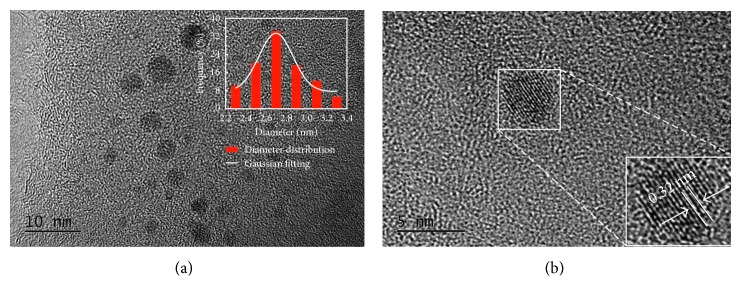
(a) TEM image of N-doped CDs. Inset shows the size distribution of N-doped CDs. (b) HRTEM reveals lattice spacing of N-doped CDs.

**Figure 2:**
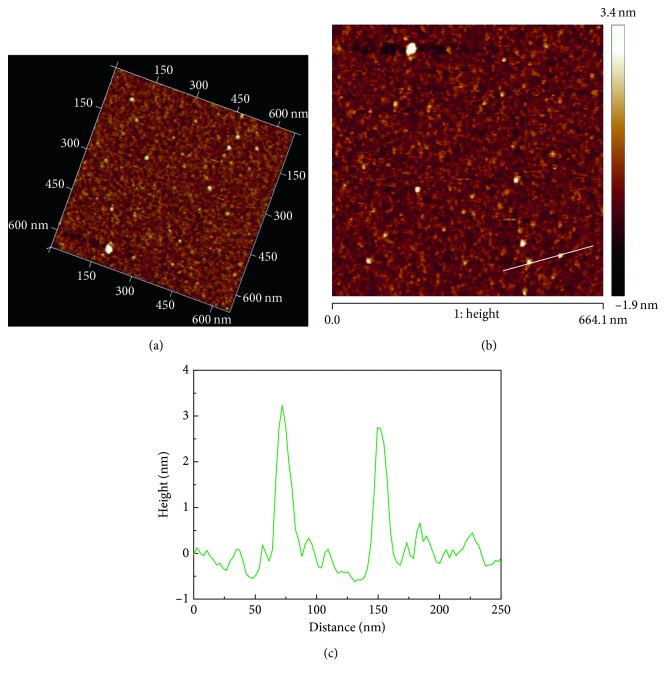
AMF images of N-doped CDs. (a) AMF 3D image, (b) AMF topography image, and (c) height profile along the line in (b).

**Figure 3:**
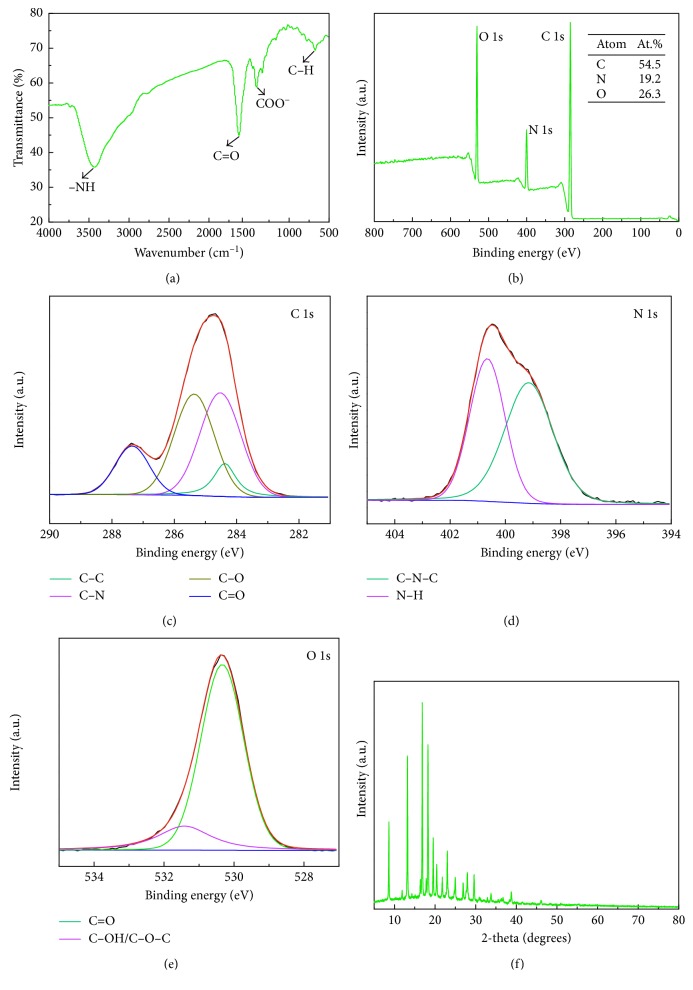
(a) FT-IR spectra of N-doped CDs. (b) XPS survey spectrum of N-doped CDs and high-resolution spectra of C1s (c), N1s (d), and O1s (e). (f) XRD pattern of N-doped CDs.

The size and morphology of N-doped CDs were characterized by HRTEM and AFM. The TEM image (Figure 1(a)) clearly revealed N-doped CDs with spherical morphology, average diameter (inset in Figure 1(a)) of 2.7 nm, and a narrow particle size distribution of 2.3–3.3 nm. HRTEM images (Figure 1(b)) showed that the average lattice spacing of the N-doped CDs was 0.32 nm, in agreement with the (002) diffraction planes of graphite [18, 19]. The AFM 3D image and topography image (Figures 2(a) and 2(b)) indicate that the N-doped CDs had a spherical shape, which is consistent with the TEM results. The average height (Figure 2(c)) of N-doped CDs was 3.16 nm, which is close to the diameter of N-doped CDs measured by TEM (2.68 nm). Hence, in accordance with the previous reports, the N-doped CDs were almost spherical carbon nanoparticles [20, 21]. FT-IR spectroscopy and XPS analyses were performed to study the chemical composition and functional groups of the N-doped CDs. In the FT-IR spectrum in Figure 3(a), the peak at 3433 cm^−1^ is attributed to the stretching vibration of –NH, the peaks at 1625 cm^−1^ indicate the existence of C=C, the peak at 1408 cm^−1^ was assigned to the bending vibration of C–NH (indicating the successful adulteration of nitrogen atoms into the C-dots), and the absorption at 674 cm^−1^ is ascribed to C–H. These FT-IR assignments were further verified by XPS analysis. XPS was used to measure the surface chemical composition and elemental analysis of N-doped CDs. The three main peaks at 284.78, 400.48, and 530.38 eV of the XPS survey spectrum (shown in Figure 3(b)) correspond to C1s, N1s, and O1s, respectively. The N-doped CDs contained 54.5 at.% carbon, 19.2 at. % nitrogen, and 26.3 at. % oxygen at the corresponding binding energies given in Figure 3(b). High-resolution XPS spectra of C1s (Figure 3(c)) can be ascribed to four component peaks with binding energies of about 284.38, 284.48, 285.38, and 287.38 eV. Here, the anterior peak located at 284.38 eV reflects the bonding structure of C–C (sp^3^) bonds, the peak located at 284.48 eV reflects the bonding structure of the C–N (sp^3^) bonds, and the peaks at 285.38 and 287.38 eV are attributed to the C–O (sp^2^) and C=O (sp^2^) bonds, respectively [22]. This indicated that the as-prepared N-doped CDs were rich in hydrophilic groups on their surfaces, which was consistent with the corresponding FT-IR spectrum. As shown in Figure 3(d) (partial XPS spectrum of N1s), the N1s peak can also be resolved into two components centred at 399.18 and 400.48 eV; the anterior peak located at 399.18 eV reflects the bonding structure of C–N–C bonds, and the second peak located at 400.48 eV reflects the bonding structure of the N–H bonds [23, 24]. The O1s peak had two components at 530.38 and 531.38 eV for adsorbed oxygen: C=O and C–OH/C–O–C (Figure 3(e)), respectively [21]. Surface functionality analyses via XPS are in agreement with FT-IR results. The above analysis indicated that the N-doped CDs synthesized might have functional groups like –COOH, –OH, and –NH. XRD patterns (Figure 3(f)) showed many narrow 2θ diffraction peaks at about 8.65°, 11.91°, 13.21°, 16.39°, 16.86°, 17.82°, 18.27°, 19.55°, 20.41°, 21.79°, 23.02°, 24.73°, 24.97°, 26.93°, 27.97°, 29.62°, 33.76°, and 38.75°, suggesting ordered carbon in N-doped CDs.

### 3.2. Spectral Properties and Cytotoxicity Assay of N-Doped CDs

**Figure 4:**
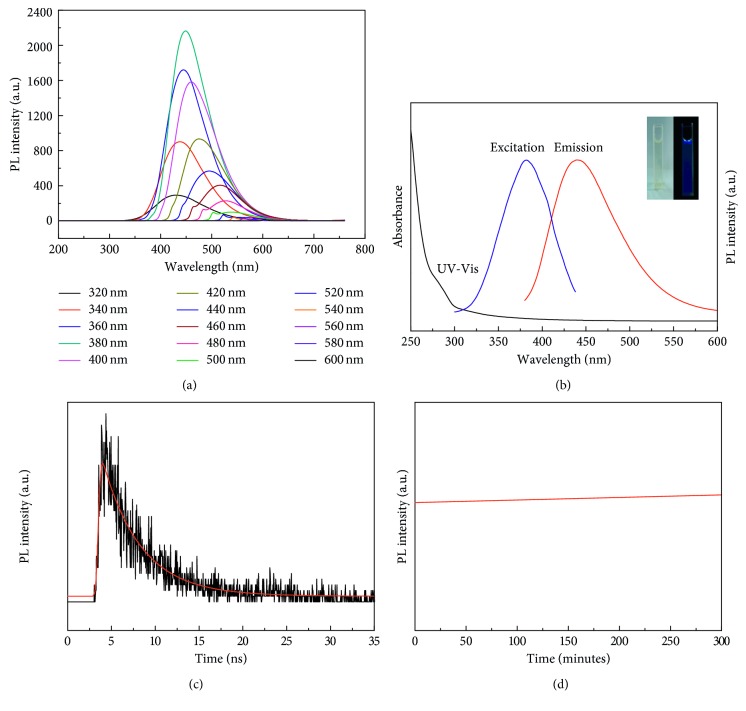
(a) UV-Vis absorption spectrum (black line), fluorescence excitation (red line, *λ*_em_ = 438 nm), and emission spectra (blue line, *λ*_ex_ = 377 nm) of N-doped CDs. The insets show the photograph (left) and fluorescence image of N-doped CD solution under 365 nm UV light (right). (b) Fluorescence emission spectra of the N-doped CDs. (c) The fluorescence decay curve of N-doped CDs. (d) Fluorescence stability of N-doped CDs.

As displayed in Figure 4(a), the excellent optical properties of the synthesized N-doped CDs were demonstrated by absorption and PL spectroscopy. The N-doped CDs displayed broad UV-Vis absorption, which was attributed to the *n*-π^*∗*^ transition in N-doped CDs. The emission wavelength of N-doped C-dots was red-shifted from 430 to 600 nm with excitation wavelength ranging from 320 to 600 nm [25]. In addition, the optimal excitation and emission wavelengths of the N-doped CDs solution were located at 377 and 438 nm (Figure 4(b)). Separately, the N-doped CD aqueous solution emitted strong blue light upon ultraviolet excitation at 365 nm (right inset, Figure 4(b)). To further investigate the optical properties of the as-obtained N-doped CDs, the PL excitation spectrum of the N-doped CDs was observed (Figure 4(b)). The spectrum displayed typical excitation wavelength dependence, and the emission wavelength was red-shifted when excited with longer wavelengths. This behaviour of the N-doped CDs has been suggested to be a result of different sizes or the existence of different emissive sites on the surfaces [26]. This excitation-dependent emission property of carbon dots has also been found in the previous reports [27–29]. We further investigated the fluorescence stability of N-doped CDs. Time-correlated single-photon counting (TCSPC) was applied to measure the fluorescence lifetime of the N-doped CDs. As presented in Figure 4(c), the decay lifetime of the N-doped CDs was measured with the previous reports [30], and the calculated average fluorescence lifetime for the N-doped CDs was 4.45 ± 0.05 ns. Moreover, the photostability (Figure 4(d)) of the N-doped CDs synthesized was tested upon continuous excitation at 360 nm for 5 h; the fluorescence remained intact without any photobleaching, which corroborates to reasonably good photostability of N-doped CDs.

**Figure 5:**
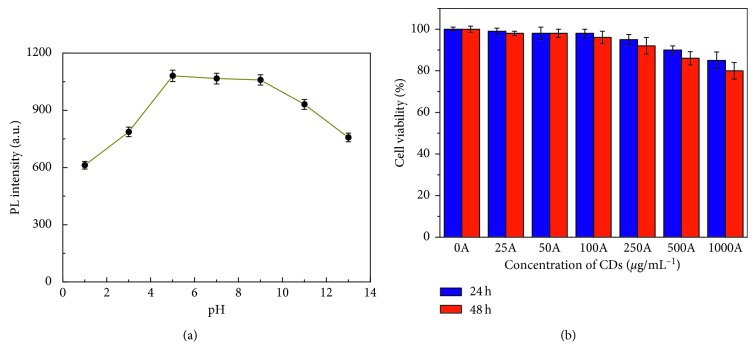
(a) PL spectra of N-doped CD aqueous solutions at different pH (*λ*_ex_ = 365 nm). (b) Cytotoxicity testing results via an MTT assay.

To investigate the applicability of N-doped CDs as a fluorescence biomarker in a practical biological environment, the fluorescence stability of N-doped CD aqueous solution was evaluated. As revealed in Figure 5(a), with increase in pH from 3.0 to 5.0, the fluorescence intensity reached a peak and decreased with pH ranging from 5.0 to 13.0. The original solution pH of the N-doped CDs was approximately 9.0. The figure shows that the fluorescence intensity of the N-doped CDs at pH 7.0–9.0 was stronger than that at 9.0 and the fluorescence intensity had not clearly declined at 7.0–9.0. A physiological environment generally has pH of 7.0–8.0, which is beneficial for bioimaging applications. In addition, fluorescence QY of the N-doped CDs was found using quinine sulphate as standard (measured at 350 nm excitation wavelength, QY = 54%). The average QY of N-doped CDs in aqueous solution at room temperature was 32.9%. The high QY is possibly due to the existence of nitrogen-containing functional groups, which are generally excellent auxochromes and greatly enhance photoluminescence.

The biological application of N-doped CDs was also explored. MTT assays were carried out to evaluate the cytotoxicity of the as-prepared N-doped CDs to living cells. As expected, cell viabilities were estimated to be greater than 90% upon addition of N-doped CDs over a wide concentration range (0–500 *µ*g/mL) and after incubation for 48 h (Figure 5(b)). High cell viabilities confirmed the low toxicity, excellent biocompatibility, and great potential of the as-prepared N-doped CDs for imaging in living cells. These also indicate that the as-prepared N-doped CDs can be considered safe for in vitro and in vivo applications.

### 3.3. Application of N-Doped CDs

#### 3.3.1. Imaging of HeLa Cells

**Figure 6:**
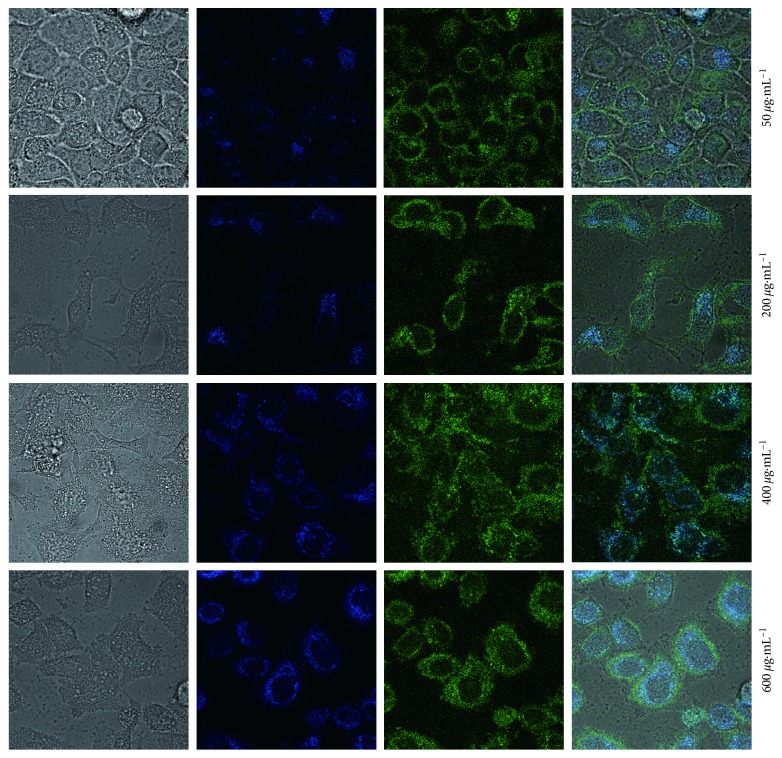
Laser scanning confocal microscopy images of HeLa cells with different concentrations of N-doped CDs.

Based on these fluorescence properties, experiments were carried out to further demonstrate the availability of the as-prepared N-doped CDs for imaging in cells and plants.  Figure 6 shows CLSM images under bright field, 405 nm, 458 nm, and 514 nm excitations of HeLa cells incubated for 4 h at 37°C with 50, 200, 400, and 600 *µ*g·mL^−1^ N-doped CDs. As shown in the figure, strong blue and green fluorescence of the HeLa cells can be seen at 405 and 488 nm. More careful observation revealed that the luminescence spots appeared widely in the membrane and cytoplasmic areas of the HeLa cells. In addition, with the increase in the concentration of N-doped CDs, the fluorescence enhancement helped identify tumour cells. According to the previous studies, the cytoplasm-specific property of N-doped CDs should be related to endocytosis [1, 9, 14, 21]. The results indicated that N-doped CDs could be used for in vitro tumour cell labelling via a simple incubation method.

#### 3.3.2. Selectivity and Ratiometric Detection of Fe^3+^ in Serum

**Figure 7:**
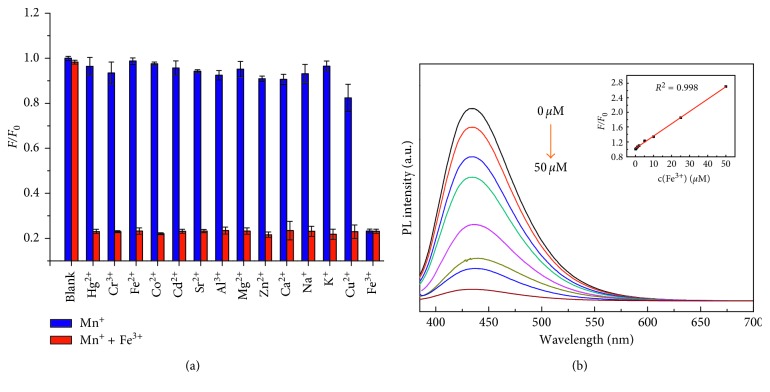
Ion detection of N-doped CDs. (a) Fluorescence recovery efficiency *F*/*F*_0_ response of the N-doped CDs toward Fe^3+^ and other interference. (b) PL spectra of N-doped CDs with different Fe^3+^ concentrations (0 to 50 *μ*M). Inset: the dependence of *F*/*F*_0_ on concentration (0 to 50 *μ*M).

As shown in Figure 7(a), under the same conditions, in sharp contrast to Fe^3+^, other ions including Hg^2+^, Cr^3+^, Fe^2+^, Co^2+^, Cd^2+^, Sr^2+^, Al^3+^, Mg^2+^, Zn^2+^, Ca^2+^, K^+^, Na^+^, and Cu^2+^ showed almost no influence on the spectra of the nanoprobe. The fluorescence could be quenched by Fe^3+^ ion due to the special coordination interaction between Fe^3+^ ion and the hydroxy groups on the surface of CDs, which may contribute to nonradiative electron transfer that involves partial transfer of an electron in the excited state to the d orbital of Fe^3+^. To further investigate the FL quenching mechanism of N-doped CDs, different concentrations of Fe^3+^ solution were added to N-doped CD stock solution. As shown in Figure 7(b), the FL intensity of N-doped CDs gradually decreased with increasing Fe^3+^ concentration and the inset shows the relationship of the relative fluorescence intensity *F*/*F*_0_ with Fe^3+^ concentration. Dynamic fluorescence quenching is described by the Stern–Volmer equation [31]. The inset in Figure 6 shows a linear relationship (*R*^2^ = 0.998) in the range of Fe^3+^ concentration from 0 to 50 *μ*M. The detection limit was 37 nM (signal-to-noise ratio of 3). The results demonstrated that N-doped CDs show promise as a sensitive and selective probe for the detection of Fe^3+^. In order to demonstrate the analytical performance of the proposed N-doped CDs in complicated biological samples, the capability of the N-doped CDs was evaluated by quantitative detection of Fe^3+^ in human serum, and it was spiked with different concentrations of Fe^3+^ and measured by the proposed method.  Table 1 shows that the recoveries were 95.2–112% with the relative standard deviation (RSD, *n* = 5) less than 5.8%, which indicates that the proposed method was sensitive and accurate.

## 4. Conclusions

We demonstrated a facile and green synthesis method to prepare novel fluorescent N-doped CDs by hydrothermal reaction using amino acid L-citrulline as precursor. The N-doped CD aqueous solution emitted strong blue light under UV irradiation with a fluorescence quantum yield of 32.9%, and the emission wavelength was red-shifted under excitation with longer wavelengths. The fluorescent N-doped CDs acted as novel fluorescence probes that facilitated simultaneous imaging of HeLa cells and sensitivity detection of Fe^3+^ ions. The N-doped CDs showed outstanding overall performance such as outstanding optical properties, good chemical and photochemical stability, inertness to interference of metal ions and biomolecular species, and excellent biocompatibility, which make N-doped CDs a desirable alternative probe for biological imaging, detection, and many other applications.

## Figures and Tables

**Table 1:** Determination of iron in human serum using N-doped CDs.

Samples	Added (*µ*mol/L)	Found (*µ*mol/L)	Recovery (%)	RSD% (*n*=5)
Serum	0.250	0.281	112	5.8
	2.50	2.61	104	4.6
	25.0	23.8	95.2	3.9

## Data Availability

The data used to support the findings of this study are included within the article, and any further information is available from the corresponding author upon request.
